# Is Trust for Sale? The Effectiveness of Financial Compensation for Repairing Competence- versus Integrity-Based Trust Violations

**DOI:** 10.1371/journal.pone.0145952

**Published:** 2015-12-29

**Authors:** Tessa Haesevoets, Chris Reinders Folmer, Alain Van Hiel

**Affiliations:** 1 Department of Developmental, Personality, and Social Psychology, Ghent University, Ghent, Belgium; 2 Erasmus School of Law, Erasmus University, Rotterdam, The Netherlands; Universidad de Alicante, ITALY

## Abstract

Despite the popularity of financial compensation as a means for addressing trust violations, the question whether (more) money can indeed buy trust back remains largely unexplored. In the present research, we focus on the role of violation type and compensation size. The results of a scenario study and a laboratory experiment show that financial compensation can effectively promote the restoration of trust for transgressions that indicate a lack of competence. Conversely, for transgressions which signal a lack of integrity, financial compensation is not an effective tool to repair trust. Moreover, our findings indicate that for both violation types, overcompensation has no positive effects on top of the impact of equal compensation. These findings therefore show that when it comes to trust, money cannot buy everything.

## Introduction

The issue of trust has been on the forefront of research agendas across a variety of disciplines in social sciences including psychology, management, organizational behavior, economics, and law [1–3]. This multidisciplinary approach highlights the pivotal role that trust plays in many aspects of our lives, as it is part of a social glue which is essential for making us the social animals that we are [4]. In fact, almost any social decision or exchange that we engage in includes some sort of trust evaluation, either towards a person, an organization, or even society as a whole. Trust thus represents a necessary ingredient to coordinate and smoothen various types of social relationships [5–6]. However, trust can easily be violated. In many situations that involve material harm, a common restorative approach for perpetrators is to offer victims a financial compensation [7–9]. In the present contribution, we examine if such financial compensations are effective for restoring trust violations that reflect a lack of competence or a lack of integrity. In addition, we investigate whether these two violation types require different levels of compensation (i.e., equal compensation or overcompensation) to effectively repair trust.

A common understanding has grown that trust is a psychological state comprising the intention to accept vulnerability based upon positive expectations of the intentions or behavior of another [3]. The presence of trust has been shown to offer numerous benefits [10]. For example, trust has been linked to love and happiness in close relationships [11]. Moreover, it also establishes effective work relationships, promotes organizational commitment and performance, positively influences cooperation, and leads to lower turnover intentions [12–16]. Although numerous researchers have focused on the positive consequences that emerge if trust is present, hardly any attention has been devoted to the psychology of moving from a state of distrust to a state of regained trust (i.e., trust restoration). This gap in the literature is regretful, particularly because people’s actions and decisions in everyday life offer numerous opportunities for violating trust (e.g., romantic betrayal or a friend who does not repay a loan) [17–20]. Acknowledging the fact that such violations lead to a host of negative outcomes in terms of emotions and behaviors [21–24], it seems crucial to develop a better understanding of how trust can be violated and if and how violated trust can be successfully repaired.

Prior research has demonstrated that when trust gets violated, people are motivated to seek explanations for the violation [25]. In this regard, a social-cognitive approach on trust has identified competence and morality beliefs as important bases for assessing trustworthiness [26]. According to this model, in order to be able to trust a person it is vital to belief that he or she is able to do what is needed (competence) and that he or she is sincere and honest (morality). A particular trust violation can often be ascribed to a violation of one of these two beliefs [18–19, 27–30]. Specifically, competence-related trust violations occur when a perpetrator violates the positive expectations that another person or group has about the perpetrator’s technical and interpersonal skills required to perform a certain task (violation of competence beliefs) [18–19, 31]. Integrity-related trust violations, in contrast, arise when a perpetrator adheres to a set of moral principles that are considered as unacceptable by another person or group, such as lying and cheating (violation of morality beliefs) [6, 18–19].

Research has revealed that competence and integrity violations are indeed distinct bases for determining trustworthiness [31–32], which reflect differently on the perpetrator. Specifically, although a single competence violation may have unpleasant consequences, it is generally not perceived as a reliable signal of a lack of competence, let alone a lack of overall reliability. Conversely, a single lapse of integrity signals the absence of general integrity, and thus automatically reflects badly on the perpetrator [18–19]. An explanation for these observations has been offered by the model of dispositional attribution of Reeder and Brewer [33]. According to this model, a single poor performance does not necessarily signal incompetence, given that both competent and incompetent people can perform poorly in certain situations; while a single dishonest behavior is considered a reliable signal of the absence of integrity, given the belief that only people of low integrity will behave in dishonest ways. Following this reasoning, the violation type (i.e., competence versus integrity) influences the victim’s beliefs in the perpetrator’s trustworthiness, and is thus likely to play a key role in determining if and how broken trust can effectively be repaired.

Previous studies of perpetrators’ attempts to restore broken trust largely focused on verbal accounts such as apologies, promises, excuses, and denials [18–20, 27–28, 34]. These studies revealed that the violation type indeed plays a crucial role in determining whether these strategies are effective to repair broken trust. Specifically, apologies are most effective after a competence-based trust violation. When the transgression reflects a lack of integrity, attributing blame to external factors by offering an excuse or a denial generates the best outcomes. However, the latter strategies pose great risks if the perpetrator’s culpability is subsequently revealed. Given that there is nothing tangible to lend credibility to such verbal response strategies, scholars have argued that they may be discounted by victims as “cheap talk” [17], and this should especially be the case when the trust violation results in monetary loss for the victim, which verbal responses do not redress. In such situations, actions may speak louder than words. Accordingly, a non-verbal response, such as the offer of a financial compensation, may be necessary to validate and strengthen the claim that the perpetrator will behave trustworthily in the future [35]. Compensations are frequently employed to address trust violations in a wide range of interpersonal and social relationships; for example, when we repay a colleague for a borrowed book that we lost or when a company reimburses a customer for a dissatisfactory product.

Prior research has indicated that financial compensation can be an effective tool for restoring a victim’s trust [7, 9, 17, 36–40]. But, does the effectiveness of financial compensation depend on the type of violation? And how much should we compensate to repair trust after such violations? Concerning this latter question, a calculative view on trust assumes that larger compensation should foster more trust [41]; but some recent studies revealed that this is not always the case. More specifically, relative to equal compensation (i.e., compensation that exactly covers the loss suffered by a victim), some studies reported positive effects of overcompensation (i.e., compensation that is greater than the loss suffered by a victim), while other studies reported neutral or even negative effects [7, 36–37, 39, 42]. In the present contribution, we test the effectiveness of financial compensation as a means to repair trust after different violation types. From a cognitive point of view, trust can be seen as a highly dynamic notion that depends on many different factors [43–46]. Considering this complexity and dynamism, it can be expected that the effectiveness of financial compensation as a means to enhance trust repair might not be straightforward, but instead depends on many other factors. One such factor may be the violation type; another may be the compensation size.

When the violation can be ascribed to a lack of competence, the wrongdoing is not indicative that the perpetrator is a bad person, because anyone can display such a low performance level under certain circumstances [18–19, 27]. Therefore, undoing the monetary loss should be sufficient to restore trust, and little benefit would arise from additional financial restitution. Hence, we hypothesize that after a competence violation, both equal compensation and overcompensation are more effective to repair trust than no compensation (Hypothesis 1a). In addition, we predict that overcompensation has no supplementary value beyond the level of equal compensation (Hypothesis 1b).

On the contrary, when the violation can be attributed to a lack of integrity, the wrongdoing signals that the perpetrator is a bad person, because only people who fall short on certain moral values will display such dishonest behavior [18–19, 27]. Therefore, we expect that only undoing the financial damage is not sufficient to restore trust. We thus hypothesize that after an integrity fault, equal compensation is not more effective than no compensation (Hypothesis 2a). With regard to the effectiveness of overcompensation, we formulate two competing hypotheses. On the one hand, after an integrity violation, the perpetrator may show his or her goodwill by going the extra mile and showing self-sacrifice by offering the victim compensation beyond the level of equal compensation. Following this rationale, overcompensation should be more effective than both no compensation and equal compensation (Hypothesis 2b). On the other hand, an integrity violation might reflect so badly on the perpetrator that even overcompensation will not be effective to repair trust. Following this perspective, overcompensation can be expected to be as ineffective as no compensation and equal compensation (Hypothesis 2c).

**Fig 1:**
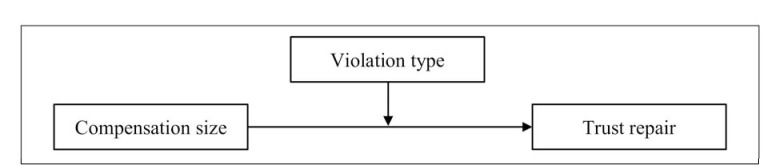
Research model.

Taken together, it can thus be predicted that the relationship between compensation size and the degree of trust repair is moderated by violation type (see Fig 1). Specifically, equal compensation and overcompensation are both expected to enhance trust repair, but only when the violation can be attributed to a lack of competence. If the violation can be ascribed to a lack of integrity, equal compensation (and possibly even overcompensation) is expected to be ineffective to establish trust repair.

## Materials and Methods

Our hypotheses were tested in two studies. Study 1 was a scenario study in which participants imagined how they would react to a perpetrator who offered no compensation, equal compensation, or overcompensation to his or her victim after inflicting a competence or an integrity violation. A key benefit of this method is that it allows the manipulation of different violation types and compensation sizes to be easily operationalized. However, because scenario studies are entirely hypothetical, we could solely measure participants trusting intentions. Moreover, an important disadvantage of this method is that reading a scenario is different from actually experiencing a specific event. Therefore, in Study 2 we conducted a laboratory experiment which allowed us to test whether our results could be cross-validated by actual trusting behavior. An advantage of lab experiments is that it is conducted in a well-controlled environment, and therefore more accurate measurements are possible. A downside of this method, however, is that it provides an artificial situation.

Both studies were conducted under the supervision of an employee of Ghent University. In accordance with the ethical protocol of Ghent University, the participants gave their written consent, and were debriefed about the purpose and content after the study. Since the present studies were in line with the “General Ethical Protocol for Scientific Research at the Faculty of Psychology and Educational Sciences of Ghent University” (www.fppw.ugent.be/FACN/AEP_engels.pdf), formal ethical approval was not needed for the conductance of these studies.

### Study 1

#### Participants and design

A total of 141 US citizens (90 men and 51 women; *M*_*age*_ = 35.43, *SD* = 10.81), recruited through Amazon Mechanical Turk, completed a scenario study in exchange for $0.5. Fourteen participants (9.9%) failed on our check questions and were thus excluded from further analyses. We employed a 2 (violation type: competence versus integrity) × 3 (compensation size: no compensation versus equal compensation versus overcompensation) between-subjects design.

#### Procedure

Participants read a short scenario which presented them with two persons: Person A and Person B. In both violation type conditions, participants were told that Person A inflicted Person B a monetary loss of $100. In the competence condition, this loss was attributed to insufficient skills on part of Person A; while in the integrity condition, the loss was ascribed to insincere behavior on part of Person A.

Subsequently, in the no compensation condition, Person A did not financially compensate Person B for the inflicted loss; in the equal compensation condition, Person A offered Person B a financial compensation of $100; and in the overcompensation condition, the compensation amounted $150.

#### Measures

Participants’ trusting intentions towards Person A were measured using the six item trust scale developed by Desmet and colleagues [7]. Particularly, we asked participants: “I trust Person A”, “I think Person A can be trusted”, “I think Person A means well for others”, “I have no trust in Person A”, “I think Person A would deceive others if he or she would benefit from it”, and “I think Person A would lie to others if he or she would gain from it” (last three items reverse-coded; 1 = *certainly not agree*, 7 = *certainly agree*). The scores on these six items were combined into a general measure of trust towards Person A (*M* = 3.05, *SD* = 1.90, α = .96).

To examine whether the violation type manipulation was successful, we asked participants: “To what extent could the financial loss be attributed to a lack of competence? (competence manipulation check)” and “To what extent could the financial loss be attributed to a lack of integrity? (integrity manipulation check)” (1 = *not at all*, 7 = *very much*). To investigate whether the compensation size manipulation was successful, we asked participants: “Did Person A offer Person B a financial compensation?” (*no* / *yes*) and “If yes, how does this compensation relate to the inflicted loss?” (1 = *compensation equals the loss*, 7 = *compensation is larger than the loss*).

### Study 2

In the second study, we investigated if our hypotheses could be confirmed when we focus on trusting behavior instead of trusting intentions. Because it is hard to spontaneously observe different trust breaches in real-life settings, we used a lab experiment to study such transgressions. Please note that in order to be able to examine participants’ actual trusting behavior following different violation types and compensation sizes, deception was necessary. That is, participants were falsely informed that they would be the observer of an ongoing situation which allegedly involved other participants, while instead all the actions and behaviors of the other players were preprogrammed. In order to limit demand characteristics, mild deception is frequently used in social psychological experiments [47–48].

#### Participants and design

A total of 137 undergraduate psychology students at Ghent University, Belgium (35 men, 102 women; *M*_*age*_ = 18.73, *SD* = 2.72), participated in a lab experiment in exchange for course credits. Again, a 2 (violation type: competence versus integrity) × 3 (compensation size: no compensation versus equal compensation versus overcompensation) between-subjects design was employed.

#### Procedure

Upon arrival to the laboratory, each participant was placed in front of a computer. Participants learned that they were connected to another classmate present in the lab, this person was referred to as Player A. Participants observed Player A during his or her interaction with another classmate, who was referred to as Player B. During this interaction, Player A violated Player B’s trust through either a competence or an integrity fault, and consequently offered this player no compensation, equal compensation, or overcompensation. To be able to manipulate these concepts, both players were preprogrammed, unbeknownst to participants.

Specifically, during the experiment participants observed Player A as he or she completed two stages of an experimental task. This task was a puzzle task in which Player A could earn money by solving mathematical puzzles [49]. During the first stage of the task, Players A and B would perform the puzzle task individually, thereby earning money for themselves. In the second stage, Players A and B would perform the puzzle task for each other, earning money for their counterpart. Participants observed Player A during both stages, thus observing his or her performance for him- or herself (in stage 1) and his or her performance for Player B (in stage 2). In this context, the violation type was manipulated, as was the level of compensation.

Player A’s level of performance during both stages constituted our manipulation of violation type. In the competence condition, participants observed Player A solving only a few puzzles in both stages (i.e., poor performance both when benefiting oneself in stage 1 and when benefiting Player B in stage 2). In the integrity condition, participants observed Player A solving all puzzles during the first stage, but only a few in the second stage (i.e., excellent performance for oneself in stage 1, poor performance for Player B in stage 2). In either case, this poor performance of Player A in the second stage meant that when the outcomes of the task were unveiled after the completion of both stages, Player A had solved less puzzles for Player B than vice versa. As a result of this poor performance, Player A had inflicted a monetary loss on Player B, as Player B received €3 less than he or she had earned for Player A.

In response to this outcome, participants observed electronic communication between the two players, in which Player A blamed the poor outcomes that he or she attained for Player B to “poor skill at this type of task”. In light of Player A’s performance for him- or herself during stage 1 (allegedly unknown to Player B, but observed by the participant), this claim was truthful in the competence condition (where Player A attained poor outcomes in both stages), making it a competence violation; but false in the integrity condition (where Player A did attain good outcomes for oneself, but not for Player B), making it an integrity violation. Note that the puzzles were equally difficult in both stages. Although making a lesser effort for someone other than oneself can be justified, lying about this makes it a clear integrity violation.

Upon completion of the task, the outcomes of both stages were unveiled, exposing Player A’s actual performance level, and the veracity of his or her claim. In response, the manipulation of compensation size was implemented, with Player A providing Player B no compensation (in the no compensation condition), a compensation of €3 (in the equal compensation condition), or a compensation of €9 (in the overcompensation condition).

#### Measures

We employed a behavioral measure of participants’ trust in Player A. Participants learned that after completion of the task, they had to take part in a second study, in which they would perform an (unrelated) dyadic task. It was explicitly stated that this additional task did not require mathematical skills. For this unrelated task, they were offered the choice between two possible interaction partners: Player A, whom they had just observed (choice which reflects trust in Person A) or Player B, the other player who was victimized and subsequently compensated by Player A (choice which reflects no trust in Person A). Specifically, we asked participants: “Which player would you prefer to complete the second study with?” (*Player A / Player B*).

To examine whether the violation type manipulation was successful, we asked participants: “To what extent shows Player A’s behavior competence? (competence manipulation check)” and “To what extent shows Player A’s behavior integrity? (integrity manipulation check)”(1 = *not at all*, 7 = *very much*). Next, to investigate whether the compensation size manipulation was successful, we asked participants: “To what extent did Player A offer Player B a lot of extra money?” (1 = *not at all*, 7 = *very much*).

## Results

### Study 1

#### Manipulation checks

We tested the effectiveness of the violation type manipulation using a 2 (violation type) × 3 (compensation size) ANOVA for both violation type manipulation checks. The results showed that participants in the competence condition attributed the violation more to a lack of competence (*M* = 6.35, *SD* = 0.85) than participants in the integrity condition (*M* = 2.41, *SD* = 1.86), *F*(1, 121) = 236.51, *p* < .001, η^2^_*p*_ = .66. Similarly, participants in the integrity condition attributed the violation more to be a lack of integrity (*M* = 6.58, *SD* = 1.04) than participants in the competence condition (*M* = 2.35, *SD* = 1.48), *F*(1, 121) = 351.81, *p* < .001, η^2^_*p*_ = .74. Moreover, a 2 (violation type) × 2 (compensation size) ANOVA on the compensation size manipulation check showed that participants in the overcompensation condition rated the compensation as larger (*M* = 6.69, *SD* = 0.68) than participants in the equal compensation condition (*M* = 1.50, *SD* = 1.52), *F*(1, 76) = 394.93, *p* < .001, η^2^_*p*_ = .84. For both the violation type manipulation checks and the compensation size manipulation check, the other main and interaction effects were non-significant (all *F*s < 2.24).

#### Trusting intentions

**Fig 2:**
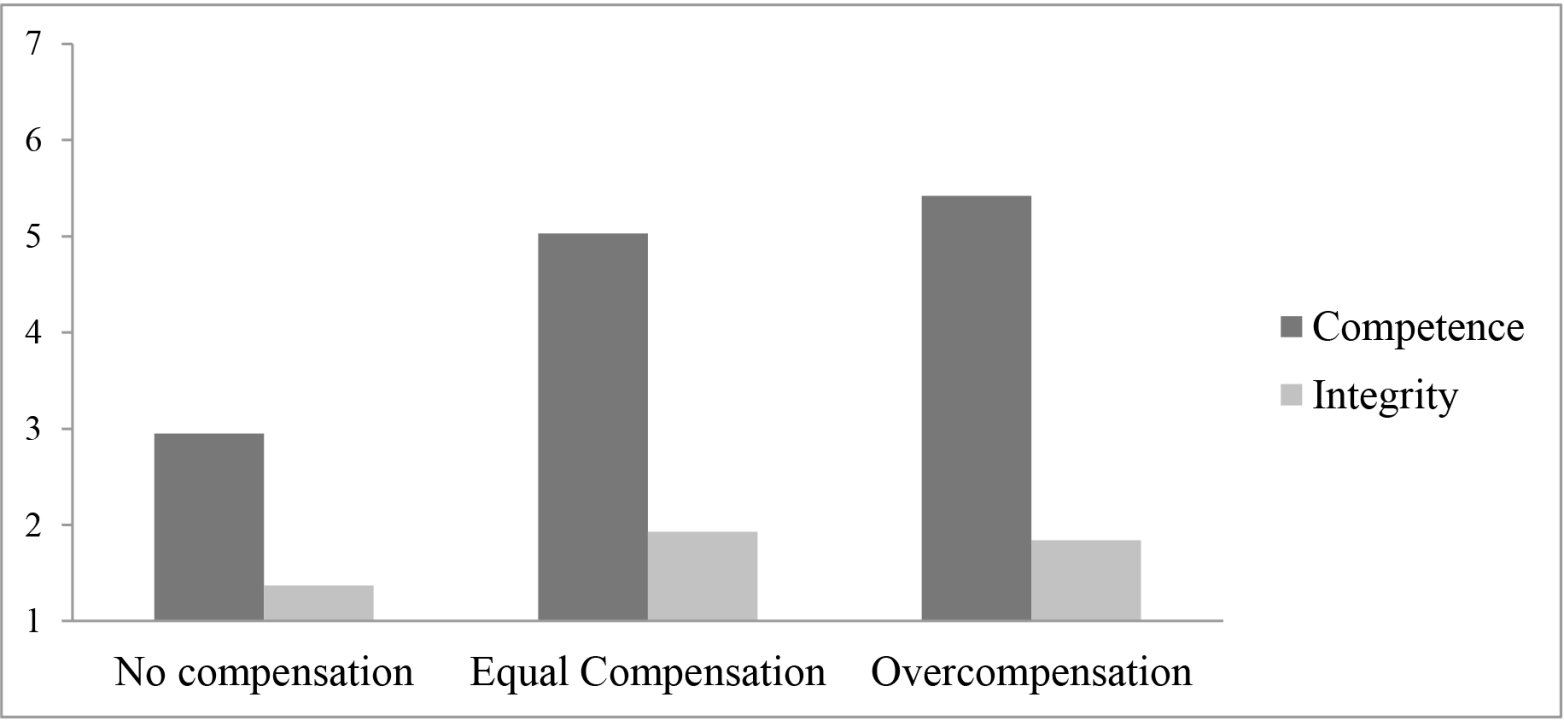
Trusting intentions as a function of violation type and compensation size.

A 2 (violation type) × 3 (compensation size) ANOVA on the trust scale showed significant main effects of violation type, *F*(1, 121) = 216.53, *p* < .001, η^2^_*p*_ = .64, and compensation size, *F*(2, 121) = 26.47, *p* < .001, η^2^_*p*_ = .30. These main effects were qualified by a significant interaction between violation type and compensation size, *F*(2, 121) = 11.10, *p* < .001, η^2^_*p*_ = .16. To test our hypotheses, this significant interaction effect was further explored using simple effects tests (with Bonferroni correction for multiple comparisons). Fig 2 visually displays this interaction term.

Within the competence condition, there was a significant effect of compensation size, *F*(2, 121) = 34.89, *p* < .001, η^2^_*p*_ = .37. As predicted by Hypothesis 1a, after a competence violation, equal compensation (*M* = 5.03, *SD* = 1.28) and overcompensation (*M* = 5.42, *SD* = 1.08) were both more effective for repairing trust than no compensation (*M* = 2.95, *SD* = 1.30; both *p*s < .001). Further, in agreement with Hypothesis 1b, the difference between overcompensation and equal compensation was non-significant after a competence violation (*p* = .709). Within the integrity condition, there is no significant effect of compensation size, *F*(2, 121) = 1.85, *p* = .162, η^2^_*p*_ = .03. In agreement with Hypothesis 2a, after an integrity violation, equal compensation (*M* = 1.93, *SD* = 0.93) was not more effective than no compensation (*M* = 1.37, *SD* = 0.62) to repair trust (*p* = .25). Moreover, as predicted by Hypothesis 2c (and opposite to the predictions made in the competing Hypothesis 2b), overcompensation (*M* = 1.84, *SD* = 0.98) was not more effective than no compensation (*p* = .416) and equal compensation (*p* > .999) to repair trust after an integrity violation.

### Study 2

#### Manipulation checks

We tested the effectiveness of our manipulations using a 2 (violation type) × 3 (compensation size) ANOVA for each manipulation check. The analysis on the competence manipulation check showed that participants in the competence condition indicated less that Player A’s behavior demonstrates competence (*M* = 3.26, *SD* = 1.39) than participants in the integrity condition (*M* = 4.72, *SD* = 1.78), *F*(1, 131) = 29.77, *p* < .001, η^2^_*p*_ = .19. Similarly, the analysis on the integrity manipulation check revealed that participants in the integrity condition indicated less that Player A’s behavior demonstrates integrity (*M* = 3.33, *SD* = 1.41) compared to participants in the competence condition (*M* = 4.14, *SD* = 0.82), *F*(1, 131) = 16.75, *p* < .001, η^2^_*p*_ = .11. Finally, the analysis on the compensation size manipulation check showed a significant main effect of compensation size, *F*(2, 131) = 27.74, *p* < .001, η^2^_*p*_ = .30. A post hoc test (Bonferroni) showed that participants indicated more often that Player A offered Player B a lot of extra money in the overcompensation condition (*M* = 6.04, *SD* = 0.87) than in the equal compensation condition (*M* = 4.76, *SD* = 1.25), as well as in the equal compensation condition compared to the no compensation condition (*M* = 3.22, *SD* = 2.76; all *p*s < .003). For all three manipulation checks, the other main and interaction effects were non-significant (all *F*s < 2.76).

#### Trusting behavior

**Fig 3:**
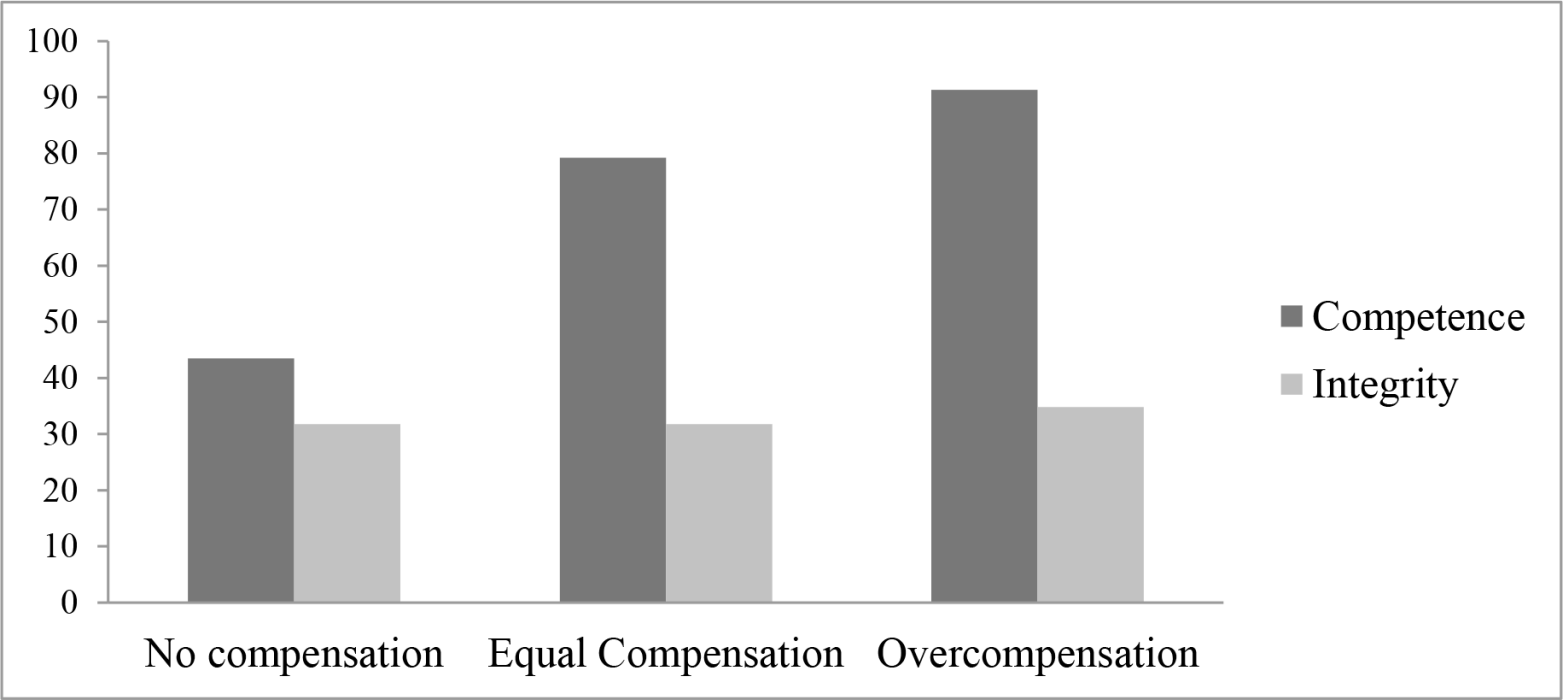
Percentage of participants who chose to complete the next task with Player A as a function of violation type and compensation size.

A logistic regression analysis with violation type, compensation size, and the interaction of violation type × compensation size as predictor variables and trusting behavior as dependent variable yielded a significant overall interaction effect (Wald = 6.24, *p* = .044). The percentages of participants who chose to complete the next task with Player A (choice which reflects trust) per condition are displayed in Fig 3. Our hypotheses predicted a specific pattern in the effectiveness of compensation size on the willingness to trust the perpetrator. In order to test these patterns we employed dummy coded variables for our compensation size variable as this is a three-level nominal variable.

Logistic regression analyses using these dummy coded variables yielded an almost significant interaction effect between violation type and the dummy that contrasted equal compensation with no compensation (Wald = 3.01, *p* = .083), a significant interaction effect between violation type and the dummy contrasting overcompensation to no compensation (Wald = 5.46, *p* = .019), and a non-significant interaction effect between violation type and the dummy that contrasted overcompensation with equal compensation (Wald = 0.65, *p* = .421). To further explore these interaction effects, we relied on simple slope analyses.

In line with Hypothesis 1a, participants in the competence condition were more inclined to trust Player A when he or she provided Player B an equal compensation compared to no compensation (*B* = 1.60, *SE* = 0.66, Wald = 5.94, *p* = .015), or an overcompensation compared to no compensation (*B* = 2.61, *SE* = 0.85, Wald = 9.43, *p* = .002). As predicted by Hypothesis 1b, the difference between equal compensation and overcompensation was non-significant after a competence violation (*B* = 1.02, *SE* = .90, Wald = 1.29, *p* = .256). Moreover, in agreement with Hypothesis 2a, participants in the integrity condition were not more inclined to trust Player A when he or she provided Player B an equal compensation compared to no compensation (*B* < 0.001, *SE* = 0.65, Wald = 0.00, *p* > .999). Finally, in accordance with Hypothesis 2c (and contrary to the predictions made in the competing Hypothesis 2b), after an integrity violation, participants were not more inclined to trust Player A when he or she provided Player B an overcompensation compared to no compensation (*B* = 0.13, *SE* = 0.63, Wald = 0.04, *p* = .833), or an overcompensation compared to an equal compensation (*B* = 0.13, *SE* = 0.63, Wald = 0.04, *p* = .833).

## Discussion

Although trust is a vital ingredient of social relationships, it is not uncommon that people violate trust, and subsequently try to restore it [18–19]. Perpetrators can try to repair broken trust by offering a financial compensation to the victim. In the present contribution, we investigated whether the positive impact of financial compensation in dependent on the violation type (competence versus integrity) and the compensation size (equal versus overcompensation). Our first study provides initial evidence that financial compensation is an effective tool to repair trusting intentions after a competence-based trust violation, but not after an integrity-based trust violation. The second study extends the results of the first study by indicating that these findings can be replicated for actual trusting behavior.

Specifically, both of our studies showed that after a competence-based trust violation, equal compensation and overcompensation are more effective for repairing trust than no compensation (Hypothesis 1a). This result is congruent with traditional justice models which suggest that when the harm is unintended − which is often the case with competence violations − financial compensation “evens the score” and is thus viewed as an appropriate way to redress the inflicted harm [50–51]. Moreover, as predicted by Hypothesis 1b, overcompensation was not more effective than equal compensation to restore trust in the aftermath of a competence violation. This finding corroborates previous research that found that once the financial harm is undone, people benefit little from additional financial restitutions [39, 42]. Thus, our studies showed that after a competence-based trust violation which resulted in a financially re-compensable harm, trust can effectively be repaired by financial compensation, and the degree of trust repair is not affected by the size of the compensation.

With regard to integrity violations, however, equal compensation is not more effective than no compensation (Hypothesis 2a). These results corroborate studies in restorative justice research which revealed that people do not consider the provision of a compensation as satisfactory when the harm is inflicted intentionally [52–53], which is mostly the case with integrity violations. With regard to the effectiveness of overcompensation, we formulated two competing hypotheses. As predicted by Hypothesis 2c (and contrary to the predictions of Hypothesis 2b) our results revealed that overcompensation is not more effective than no compensation and equal compensation. Our results thus extend restorative justice research by showing that in response to an integrity-based trust violation, overcompensation is also not effective in repairing trust. Hence, our reasoning that after an integrity violation, the perpetrator should show his or her goodwill by going the extra mile by offering the victim compensation beyond the level of equal compensation has not been sustained. Rather, our findings indicate that in the aftermath of an integrity fault, money is unsuccessful to restore trust.

In sum, our findings are in line with the cognitive view on trust which holds that the presence of trust is a result of a complex interplay between many different factors [43–46]. More specifically, our results confirm the complexity of the trust concept by showing that the effectiveness of financial compensation as a means to repair trust is not straightforward but rather depends on whether the violation can be attributed to a lack of competence or a lack of integrity.

An important strength of the present research is that it provides an experimental method by means of which competence and integrity violations can experimentally be induced in a lab context. Thereby, it provides an important contribution to the trust literature, which until now has solely relied on scenarios to understand the impact of violation type on the restoration of trust. Our research thus provides a much needed and easy applicable paradigm that can actually manipulate violation type experimentally. Moreover, this method can also be employed to study how victims, rather than observers, may respond to trust repair strategies after competence and integrity violations. While this perspective has been overlooked in extant research on such violations [18–19, 27], it can provide crucial insight into how such reparations may affect the relationship between victim and offender, rather than the community at large [38, 54]. The present research provides a crucial tool to study this question, and we regard this as a highly valuable avenue for future research.

A limitation of the present research, however, is that we only focused on situations in which the harm was financially compensable. It could be expected that in other contexts − in which the loss is framed in non-financial terms–financial compensation will even be less effective to promote trust repair. Future research should thus consider the context in which trust is impaired. However, it is important to note that despite the compensation friendly context in our studies, compensation size effects (of equal compensation versus overcompensation) failed to occur.

## Supporting Information

S1 Datafile Raw data Study 1. Data file with raw data of Study 1.
(TXT)

S2 Datafile Raw data Study 2. Data file with raw data of Study 2.
(TXT)

S1 Dataset Recoded data Study 1. Dataset with recoded data of Study 1.
(SAV)

S2 Dataset Recoded data Study 2. Dataset with recoded data of Study 2.
(SAV)

S1 Questions Questions Study 1. Questions given to participants in Study 1.
(DOCX)

S2 Questions Questions Study 2. Questions given to participants in Study 2.
(DOCX)

S1 Scenarios Scenarios Study 1. Scenarios given to participants in Study 1.
(DOCX)

S1 Syntax Syntax Study 1. Syntax file of Study 1.
(SPS)

S2 Syntax Syntax Study 2. Syntax file of Study 2.
(SPS)

## References

[1] Dirks KT, Lewicki RJ, Zaheer A. Repairing relationships within and between organizations: Building a conceptual foundation. Academy of Management Review 2009; 34: 68–84.

[2] Kramer RM, Lewicki RJ. Repairing and enhancing trust: Approaches to reducing organizational trust deficits. Academy of Management Annals 2010; 4: 245–277.

[3] Rousseau D, Sitkin S, Burt R, Camerer C. Not so different after all: A cross-discipline view of trust. Academy of Management Review 1998; 23: 393–404.

[4] Van Vugt M, Hart C. Social identity as social glue: The origins of group loyalty. Journal of Personality and Social Psychology 2004; 86: 585–598. 15053707 10.1037/0022-3514.86.4.585

[5] De Cremer D, Desmet PTM. Restoring trust depends on the victim’s motives: A motivated trust repair model In: Kramer RM, Pittinsky T, editors. Restoring trust: Challenges and prospects. NY: Oxford University Press; 2012 pp. 241–256.

[6] Mayer RC, Davis JH, Schoorman FD. An integrative model of organizational trust: Past, present, and future. Academy of Management Review 2007; 32: 344–354.

[7] Desmet PTM, De Cremer D, Van Dijk E. Trust recovery following voluntary or forced financial compensations in the trust game: The role of trait forgiveness. Personality and Individual Differences 2011; 51: 267–273.

[8] Davidow M. Organizational responses to customer complaints: What works and what doesn’t. Journal of Service Research 2003; 5: 225–250.

[9] Worsfold K, Worsfold J, Bradley G. Interactive effects of proactive and reactive service recovery strategies: The case of rapport and compensation. Journal of Applied Social Psychology 2007; 37: 2496–2517.

[10] Dirks KT, Ferrin DL. Trust in leadership: Meta-analytic findings and implications for organizational research. Journal of Applied Psychology 2002; 87: 611–628. 12184567 10.1037/0021-9010.87.4.611

[11] Rempel JK, Holmes JG, Zanna MP. Trust in close relationships. Journal of Personality and Social Psychology 1985; 49: 95–112. 11474726

[12] Costigan RD, Insinga RC, Berman JJ, Kranas G, Kureshov VA. A four-country study of the relationship of affect-based trust to turnover intention. Journal of Applied Social Psychology 2012; 42: 1123–1142.

[13] De Cremer D, Tyler TR (2005). Managing group behavior: The interplay between procedural fairness, self, and cooperation In: Zanna M, editor. Advances in Experimental Social Psychology. New York: Academic Press; 2005 pp.151–218.

[14] Dirks KT, Ferrin DL. The role of trust in organizational settings. Organization Science 2001; 12: 450–467.

[15] Lee D, Stajkovic AD, Cho B. Interpersonal trust and emotion as antecedents of cooperation: Evidence from Korea. Journal of Applied Social Psychology 2011; 41: 1603–1631.

[16] Sousa-Lima M, Michel JW, Caetano A. Clarifying the importance of trust in organizations as a component of effective work relationships. Journal of Applied Social Psychology 2013; 43: 418–427.

[17] Bottom WP, Gibson K, Daniels S, Murnighan JK. When talk is not cheap: Substantive penance and expressions of intent in rebuilding cooperation. Organization Science 2002; 13: 497–513.

[18] Kim PH, Dirks KT, Cooper CD, Ferrin DL. When more blame is better than less: The implications of internal vs. external attributions for the repair of trust after a competence- vs. integrity-based trust violation. Organizational Behavior and Human Decision Processes 2006; 99: 49–65.

[19] Kim PH, Ferrin DL, Cooper CD, Dirks KT. Removing the shadow of suspicion: The effects of apology versus denial for repairing competence- versus integrity-based trust violations. Journal of Applied Psychology 2004; 89: 104–118. 14769123 10.1037/0021-9010.89.1.104

[20] Schweitzer M, Hershey J, Bradlow E. Promises and lies: Restoring violated trust. Organizational Behavior and Human Decision Processesn 2006; 101: 1–19.

[21] Joskowicz-Jabloner L, Leiser D. Varieties of trust-betrayal: Emotion and relief patterns in different domains. Journal of Applied Social Psychology 2013; 43: 1799–1813.

[22] Pillutla MM, Murnighan JK. Unfairness, anger, and spite: Emotional rejections and ultimatum offers. Organizational Behavior and Human Decision Processes 1996; 68: 208–224.

[23] Bies RJ, Tripp TM. Beyond Distrust: “Getting Even” and the Need for Revenge In: Kramer RM, Tyler TR, editors. Trust in Organizations: Frontiers of Theory and Research. Thousand Oaks, CA: Sage; 1996 pp. 246–260.

[24] Haden SC, Hojjat M. Aggressive responses to betrayal: Type of relationship, victim’s sex, and nature of aggression. Journal of Social and Personal Relationships 2006; 23: 101–116.

[25] Blount S. When social outcomes aren’t fair: The effect of causal attributions on preferences. Organizational Behavior and Human Decision Processes 1995; 63: 131–144.

[26] Castelfranchi C, Falcone R (2005). Socio-cognitive theory of trust In: Pitt J, editor. Open Agent Societies: Normative Specifications in Multi-Agent. London: Wiley; 2005 pp.58–89.

[27] Ferrin DL, Kim PH, Cooper CD, Dirks KT. Silence speaks volumes: The effectiveness of reticence in comparison to apology and denial for responding to integrity- and competence-based trust violations. Journal of Applied Psychology 2007; 92: 893–908. 17638453 10.1037/0021-9010.92.4.893

[28] Kim PH, Dirks KT, Cooper CD. The repair of trust: A dynamic bilateral perspective and multilevel conceptualization. Academy of Management Review 2009; 34: 401–422.

[29] Janowicz-Panjaitan MK, Krishnan R. Measures for dealing with competence and integrity violations of interorganizational trust at the corporate and operating levels of organizational hierarchy. Journal of Management Studies 2009; 46: 245–268.

[30] Xie Y, Peng S. How to repair customer trust after negative publicity: The roles of competence, integrity, benevolence, and forgiveness. Psychology and Marketing 2009; 26: 572–589.

[31] Butler JK Jr, Cantrell RS. A behavioral decision theory approach to modeling dyadic trust in superiors and subordinates. Psychological Reports 1984; 55: 19–28.

[32] Barber B. The logic and limits of trust. New Brunswick, NJ: Rutgers University Press; 1983.

[33] Reeder GD, Brewer MB. A schematic model of dispositional attribution in interpersonal perception. Psychological Review 1979; 86: 61–79.

[34] Tomlinson EC, Dineen BR, Lewicki RJ. The road to reconciliation: Antecedents of victim willingness to reconcile following a broken promise. Journal of Management 2004; 30: 165–187.

[35] Dirks KT, Kim PH, Ferrin DL, Cooper CD. Understanding the effects of substantive responses on trust following a transgression. Organizational Behavior and Human Decision Processes 2011; 114: 87–103.

[36] Desmet PTM, De Cremer D, Van Dijk E. On the psychology of financial compensations to restore fairness transgressions: When intentions determine value. Journal of Business Ethics 2010; 95: 105–115.

[37] Desmet PTM, De Cremer D, Van Dijk E. In money we trust? The use of financial compensations to repair trust in the aftermath of distributive harm. Organizational Behavior and Human Decision Processes, 2011; 114: 75–86.

[38] Haesevoets T, Reinders Folmer C, Van Hiel A. More money, more trust? Target and observer differences in the effectiveness of financial overcompensation to restore trust. Psychologica Belgica 2014; 54: 389–394. 10.5334/pb.ay PMC5854215 30479410

[39] Haesevoets T, Van Hiel A, Reinders Folmer C, De Cremer D. What money can’t buy: The psychology of financial overcompensation. Journal of Economic Psychology 2014; 42: 83–95.

[40] DeCarufel A. Victims’ satisfaction with compensation: Effects of initial disadvantage and third party intervention. Journal of Applied Social Psychology 1981; 11: 445–459.

[41] Lewicki RJ, Wiethoff C, Tomlinson E (2005). What is the role of trust in organizational justice? In: Greenberg J, Colquitt J, editors. Handbook of organizational justice. Mahwah, NJ: Lawrence Erlbaum Associates; 2005 pp. 247–270.

[42] Haesevoets T, Reinders Folmer C, De Cremer D, Van Hiel A. Money isn’t all that matters: The use of financial compensation and apologies to preserve relationships in the aftermath of distributive harm. Journal of Economic Psychology 2013; 35: 95–107.

[43] Castelfranchi C, Falcone R, Marzo F. Being trusted in a social network: Trust as relational capital. In Stølen K, Winsborough WH, Martinelli F, Massacci F, editors. Trust Management. Berlin, Heidelberg: Springer; 2006 pp. 19–32.

[44] Falcone R, Castelfranchi C. Social trust: A cognitive approach In: Castelfranchi C, Tan YH, editors. Trust and deception in virtual societies (pp. 55–90). Netherlands: Springer, 2001. pp. 55–90.

[45] Falcone R, Singh M, Tan YH. Introduction: Bringing together humans and artificial agents in cyber-societies: A New field of trust research In: Falcone R, Singh M, Tan YH, editors. Trust in Cyber-societies. Berlin, Heidelberg: Springer; 2001 pp. 1–7.

[46] Marzo F, Castelfranchi C. Trust as individual asset in a network: a cognitive analysis In: Spagnolett P., editor. Organizational Change and Information Systems. Berlin, Heidelberg: Springer; 2013 pp. 167–175.

[47] Hewstone M, Stroebe W, Jonas K (2012). An Introduction to social psychology. Chichester: BPS Blackwell.

[48] Van Hiel A (2013). Sociale Psychologie. Gent: Academia Press.

[49] Mazar N, Amir O, Ariely D. The dishonesty of honest people: A theory of self-concept maintenance. Journal of Marketing Research 2008; 45: 633–644.

[50] Austin W, Walster E, Utne M. Equity and the law: The effect of a harmdoer’s ‘suffering in the act’ on liking and assigned punishment In: Berkowitz L, Walster E, editors. Advances in experimental social psychology. New York: Academic Press; 1976 pp. 163–190.

[51] Brickman P. Crime and punishment in sports and society. Journal of Social Issues 1997; 33: 140–164.

[52] Darley JM, Pittman TS. The psychology of compensatory and retributive justice. Personality and Social Psychology Review 2003; 7: 324–336. 14650389 10.1207/S15327957PSPR0704_05

[53] Tyler TR, Boeckmann RJ, Smith HJ, Huo YJ. Social justice research in a diverse society. Boulder, CO: Westview Press, 1997.

[54] Risen JL, Gilovich T. Target and observer differences in the acceptance of questionable apologies. Journal of Personality and Social Psychology 2007; 92: 418–433. 17352601 10.1037/0022-3514.92.3.418

