# Supplementary material for: Is Trust for Sale? The Effectiveness of Financial Compensation for Repairing Competence- versus Integrity-Based Trust Violations
Source: PLoS One. 2015 Dec 29;10(12):e0145952. doi: 10.1371/journal.pone.0145952 (PMC4694657; doi:10.1371/journal.pone.0145952)
Supplement: S1 Questions — Questions given to participants in Study 1. (DOCX) [file pone.0145952.s005.docx]

**QUESTIONS GIVEN TO PARTICIPANTS IN STUDY 1**

| **VARIABLE** | **QUESTION** | **RESPONSE OPTIONS** |
| --- | --- | --- |
| AGE | Age? | (open question) |
| SEX | Gender? | Male / Female |
| TRUST_01 | I trust Person A. | 1 = Certainly not agree,  7 = Certainly agree |
| TRUST_02 | I have no trust in Person A. | 1 = Certainly not agree,  7 = Certainly agree |
| TRUST_03 | I think Person A would deceive others if (s)he would benefit from it. | 1 = Certainly not agree,  7 = Certainly agree |
| TRUST_04 | I think Person A can be trusted. | 1 = Certainly not agree, 7 = Certainly agree |
| TRUST_05 | I think this Person A would lie to others if (s)he would gain from it. | 1 = Certainly not agree, 7 = Certainly agree |
| TRUST_06 | I think this Person A means well for others. | 1 = Certainly not agree,  7 = Certainly agree |
| MANCHECK_COMPETENCE | To what extent could the financial loss be attributed to a lack of competence? | 1 = Not at all,  7 = Very much |
| MANCHECK_INTERGRITY | To what extent could the financial loss be attributed to a lack of integrity? | 1 = Not at all,  7 = Very much |
| MANCHECK_COMPENSATION | How does this compensation relate to the inflicted loss? | 1 = Compensation equals the loss,  7 = Compensation is larger than the loss |
| CHECK_VIOLATION | The financial loss can be ascribed to? | 1 = A lack of competence  2 = A lack of integrity |
| CHECK_COMPENSATION | Did Person A offer Person B a financial compensation? | 1 = No  2 = Yes, a compensation that equals the damage  3 = Yes, a compensation that is larger than the damage |
| CHECK_01&02 | Please select the … response box. (attention checks) | 1 = Correct response,  0 = Incorrect response |
