# Supplementary material for: Is Trust for Sale? The Effectiveness of Financial Compensation for Repairing Competence- versus Integrity-Based Trust Violations
Source: PLoS One. 2015 Dec 29;10(12):e0145952. doi: 10.1371/journal.pone.0145952 (PMC4694657; doi:10.1371/journal.pone.0145952)
Supplement: S2 Questions — Questions given to participants in Study 2. (DOCX) [file pone.0145952.s006.docx]

**QUESTIONS GIVEN TO PARTICIPANTS IN STUDY 2**

| **VARIABLE** | **QUESTION ASKED TO PARTICIPANTS** | **RESPONSE OPTIONS** |
| --- | --- | --- |
| SEX | Age? | (open question) |
| AGE | Gender? | Male / Female |
| TRUST_BEHAVIOR | Which player would you prefer to complete the second study with? | Player A / Player B |
| MANCHECK_COMPETENCE | To what extent shows Player A’s behavior competence? | 1 = Not at all, 7 = Very much |
| MANCHECK_INTEGRITY | To what extent shows Player A’s behavior integrity? | 1 = Not at all, 7 = Very much |
| MANCHECK_COMPENSATION | To what extent did Player A offer Player B a lot of extra money? | 1 = Not at all, 7 = Very much |
