# Supplementary material for: Is Trust for Sale? The Effectiveness of Financial Compensation for Repairing Competence- versus Integrity-Based Trust Violations
Source: PLoS One. 2015 Dec 29;10(12):e0145952. doi: 10.1371/journal.pone.0145952 (PMC4694657; doi:10.1371/journal.pone.0145952)
Supplement: S1 Scenarios — Scenarios given to participants in Study 1. (DOCX) [file pone.0145952.s007.docx]

**SCENARIOS GIVEN TO PARTICIPANTS IN STUDY 1**

*---*

*Please try to imagine the situation below as vividly as possible.*

**[VIOLATION TYPE MANIPULATION]**

[COMPETENCE VIOLATION]

People can inflict financial harm to others because they have insufficient skills and abilities. Suppose that, due to a lack of competence on part of Person A, Person B has suffered a financial loss of 100 dollar.

[INTEGRITY VIOLATION]

People can inflict financial harm to others because they are dishonest and insincere. Suppose that, due to a lack of honesty on part of Person A, Person B has suffered a financial loss of 100 dollar.

*---*

*Below, you find Person A’s reaction to this incident.*

**[COMPENSATION SIZE MANIPULATION]**

[NO COMPENSATION]

In the aftermath of this incident, Person A offered Person B no financial compensation.

[EQUAL COMPENSATION]

In the aftermath of this incident, Person A offered Person B a financial compensation of 100 dollar. This compensation thus equals the loss suffered by Person B.

[OVERCOMPENSATION]

In the aftermath of this incident, Person A offered Person B a financial compensation of 150 dollar. This compensation is thus larger than the loss suffered by Person B.

---
